# Role of membrane compartment occupied by Can1 (MCC) and eisosome subdomains in plant pathogenicity of the necrotrophic fungus *Alternaria brassicicola*

**DOI:** 10.1186/s12866-019-1667-4

**Published:** 2019-12-16

**Authors:** Justine Colou, Guillaume Quang N’Guyen, Ophélie Dubreu, Kévin Fontaine, Anthony Kwasiborski, Franck Bastide, Florence Manero, Bruno Hamon, Sophie Aligon, Philippe Simoneau, Thomas Guillemette

**Affiliations:** 1 Institut de Recherche en Horticulture et Semences - UMR 1345, INRA, Université d’Angers, Agrocampus-Ouest, SFR 4207 QuaSaV, 42 rue Georges Morel, 49071 Beaucouzé Cedex, Angers, France; 2 Institut de Biologie Intégrative et des Systèmes, Département de Biologie, PROTEO, Université Laval, Pavillon Charles-Eugène-Marchand, 1030 Avenue de la Médecine, QC, Québec G1V 0A6 Canada; 3 ANSES, Laboratoire de la Santé des Végétaux, Unité de Mycologie, Domaine de Pixérécourt, 54220 Malzéville, France; 4 Plateforme SCIAM, Institut de Biologie en Santé, CHU, Université d’Angers, 4, Rue Larrey, 49933 Angers Cedex, France

**Keywords:** Eisosome, Fungi, Plant pathogen, Appressoria, Seed, Plasma membrane

## Abstract

**Background:**

MCC/eisosomes are membrane microdomains that have been proposed to participate in the plasma membrane function in particular by regulating the homeostasis of lipids, promoting the recruitment of specific proteins and acting as provider of membrane reservoirs.

**Results:**

Here we showed that several potential MCC/eisosomal protein encoding genes in the necrotrophic fungus *A. brassicicola* were overexpressed when germinated spores were exposed to antimicrobial defence compounds, osmotic and hydric stresses, which are major constraints encountered by the fungus during the plant colonization process. Mutants deficient for key MCC/eisosome components did not exhibit any enhanced susceptibility to phytoalexins and to applied stress conditions compared to the reference strain, except for a slight hypersensitivity of the *∆∆abpil1a-abpil1b* strain to 2 M sorbitol. Depending on the considered mutants, we showed that the leaf and silique colonization processes were impaired by comparison to the wild-type, and assumed that these defects in aggressiveness were probably caused by a reduced appressorium formation rate.

**Conclusions:**

This is the first study on the role of MCC/eisosomes in the pathogenic process of a plant pathogenic fungus. A link between these membrane domains and the fungus ability to form functional penetration structures was shown, providing new potential directions for plant disease control strategies.

## Background

The plasma membrane (PM) acts as a protective barrier for all living cells and mediates a variety of dynamic processes, including nutrient uptake, endocytosis, exocytosis, cell wall biogenesis or signal transduction [1–3]. To achieve these processes, the PM is highly compartmentalized consisting of distinct subdomains with different protein / lipid environments and specialized functions. In yeast, several large membrane compartments have been characterized: MCC (Membrane Compartment of Can1, arginine permease), MCT (Membrane Compartment of TORC2, target of rapamycin kinase complex 2) and MCP (Membrane Compartment Pma1, acting as an H+ plasma membrane ATPase) [4–7]. More recently, another distinct punctate compartment was identified and called Membrane Compartment occupied by Wsc1 (MCW), which plays an essential role for cell wall integrity pathway activation [8].

The MCC domains have been found only in fungal cells, microalgae and lichens [9]. They were associated with peripheral membrane proteins located on the cytoplasmic side forming a complex termed eisosome [10]. Together, MCCs and eisosomes form a stable and immobile PM structure associated with membrane invaginations that are about 300 nm long and 50 nm deep furrows [11]. Contrary to MCP, MCC/eisosomes probably do not have a direct role in dynamic processes such as endocytosis and secretion, since eisosome proteins do not colocalize with actin patches or sites of endocytosis [4–6, 12]. Membrane protein constituents of MCCs include several proton symporters, such as Can1 (H+/arginin symporter), Fur4 (H+/uracile symporter), and Tat2 (H+/tryptophan or H+/tyrosine symporter) [13, 14], as well as members of different families of tetraspan proteins that are known to have four transmembrane domains [14–16]. The first family of tetraspanners found in MCCs includes Sur7 and the paralogous proteins Fmp45, Pun1, and Ynl194c. The second family of tetraspanners contains Nce102 and Fhn1. Pil1 and its paralog Lsp1 in yeast, or their homologous Pil(1)A / Pil(1)B in filamentous fungi, are the most abundant proteins in eisosomes. These two proteins are essential for the membrane curvature and formation of furrows by assembling into filaments and binding membrane through their BAR (Bin/Amphiphysin/Rvs) domains [17, 18]. In yeast, Pil1 and Lsp1 are regulated by a pair of redundant protein kinases Pkh1 and Pkh2 mediating the long-chain-base-signaling pathway. However, the impact of these phosphorylation/dephosphorylation on the MCC/eisosome structure is still unclear [19–23].

MCC/eisosomes have been proposed to participate in the plasma membrane function by regulating the lipid homeostasis, in particular sphingolipids and phosphatidylinositol 4,5- bisphosphate (PI4,5P_2_) [16, 24, 25]. Moreover, due to their specific lipid composition, they would promote the recruitment of specific proteins and their subsequent protection from endocytosis [14, 26] and also would act as provider of membrane reservoirs required for membrane expansion in response to particular stresses [27, 28]. Mutations targeting MCC/eisosome proteins in yeasts and filamentous fungi can result in abnormal hyphal morphogenesis and promote other defects in endocytosis, autophagic processes, cell wall synthesis or protection against stresses [2, 18, 29]. Moreover, MCC/eisosomes have been found to contribute to pathogenicity of *Candida albicans* and *Beauveria bassiana*, infecting humans and arthropods, respectively [29–32]. Several factors may explain the virulence reduction in MCC/eisosome mutants. First, in *C. albicans*, *sur7* and *nce102* mutants were defective in forming hyphal filaments that promote invasive growth into tissues. MCC/eisosome mutants were also generally found to be more sensitive to a variety of stresses and to host immune response encountered in vivo [33]. Finally, in *B. bassiana,* all single/double deletion mutants of *pil1A* and *pil1B* resulted in reduced ability to secrete Pr1 proteases required for host cuticle degradation [29]. More recently, [34] showed that deletion of Sfp2, which is a tetraspan protein (more closely related to yeast Pun1 than to Sur7), resulted in strong defects in polarized growth, cell wall integrity and endocytosis in the mycoparasitic fungus *Trichoderma atroviride*. The deletion or overexpression of *spf2* also significantly altered mycoparasitic activity of *T. atroviride* as well as the transcriptional regulation of chitin synthase and chitinase-encoding genes [34].

In this study, we investigated the importance of the MCC/eisosomes with respect to the ability of the plant necrotrophic fungus *Alternaria brassicicola* to efficiently accomplish key steps of its pathogenic life cycle. *A. brassicicola* is an economically important seed-borne pathogen of Brassicaceae species causing black spot disease on aerial parts of the plants, including siliques, seeds and stems. Its transmission to seeds is a major component of pathogen fitness promoting the dispersal and long-term survival of the fungus [35]. Here, we first identified the major components of the MCC/eisosomes in the *A. brassicicola* genome and followed their gene expression in response to various stresses encountered by the fungal pathogen during the infection process of its host. Then, we investigated the role of major MCC/eisosome components on pathogenicity by phenotyping deficient mutants.

## Results

### Identification of a. brassicicola MCC/eisosome components

**Fig. 1:**
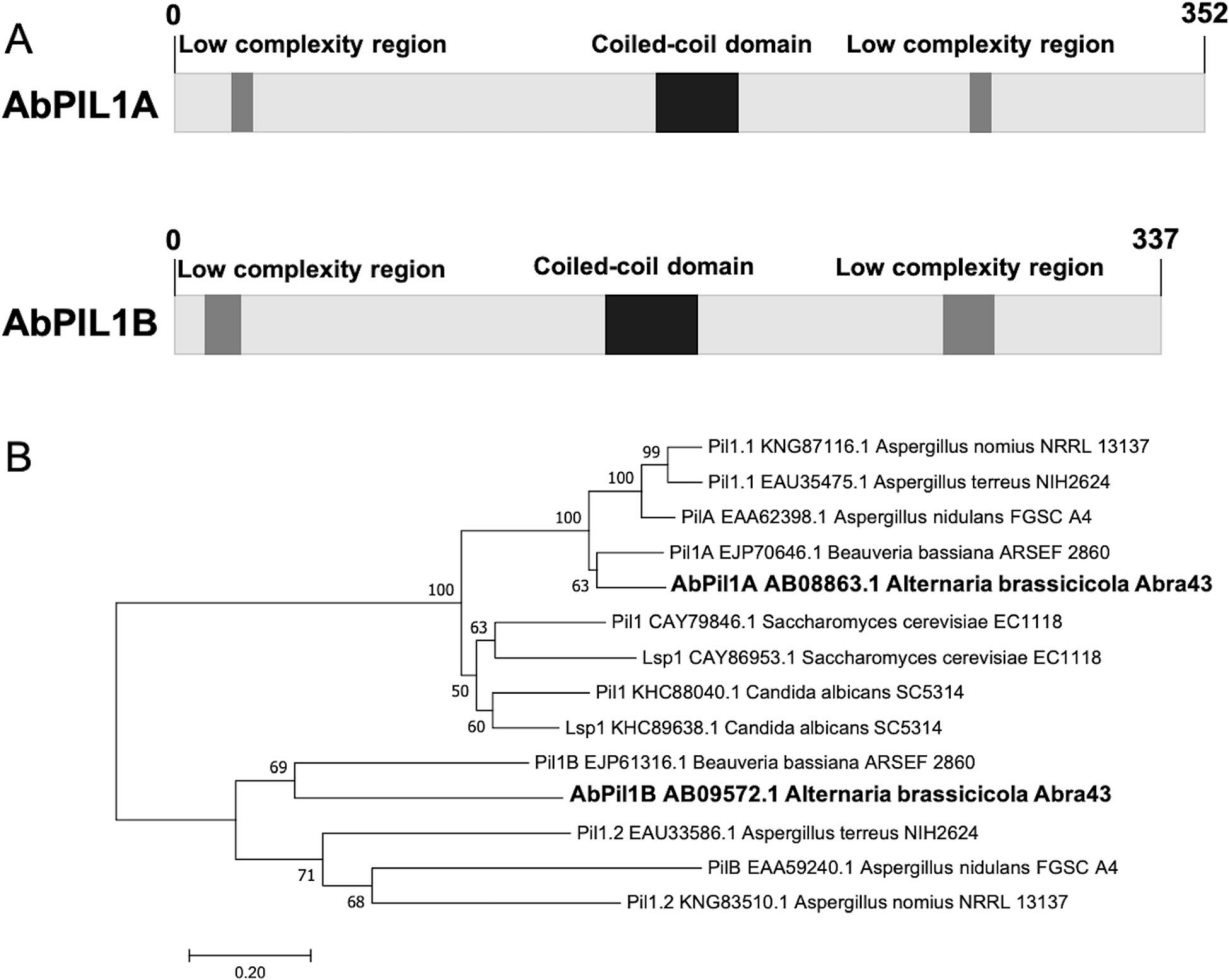
Bioinformatic features of PIL1A and PIL1B homologues in *A. brassicicola*. **a** Conserved domains predicted from the respective sequences of AbPIL1A and AbPIL1B using SMART software at http://smart.emblheidelberg.de/. **b** Phylogenetic tree for AbPIL1 and LSP1 homologs found in *B. bassiana* (Bba), *Aspergillus* species (Ate: *A. terreus*; Ano: *A. nomius*; and Ani: *A. nidulans* and yeasts (Cal: *Candida albicans*; Cor: *C. orthopsilosis*; and Sce: *S. cerevisiae*). The phylogenetic analysis was based on the neighbor-joining method in MEGA7 software at http://www.megasoftware.net/. Bootstrap values of 1000 replications are given at nodes

From the interactive JGI fungal portal MycoCosm (http://genome.jgi.doe.gov/Altbr1/Altbr1.home.html) *A. brassicicola* proteome, we searched for homologous proteins of the main *Saccharomyces cerevisiae* MCC/eisosome proteins, based on the protein set reported by [2]. We identified ten *A. brassicicola* eisosomal proteins and six others MCC proteins (Table 1). Two *A. brassicicola* proteins (AB08863 and AB09572, called AbPIL1A and AbPIL1B, respectively) are homologous to yeast Pil1 and Lsp1, and are 50.8% identical in sequence. AbPIL1A and AbPIL1B proteins are 353 and 431 amino acids long, respectively, and both contain an arfaptin homology (AH)/ Bin-Amphiphysin-Rvs (BAR) domain, which consists of three alpha-helices. As described by [29, 36], each sequence harbors the typical central coiled-coil domain and low complexity regions located on each side at N-terminus and C-terminus (Fig. 1). Phylogenic analysis showed that AbPIL1A was distinct from yeast Pil1/Lsp1 homologues and was grouped with Pil1A proteins from *B. bassiana* and *aspergilli* (Fig. 1). As expected, AbPIL1B sequence fell into the Pil1B clade. Concerning membrane proteins that are constituents of MCCs, only one homologous protein to yeast Sur7 and Nce102, respectively, was found in the *A. brassicicola* genome. While three putative members of the Sur7 family of proteins were present in *Aspergillus nidulans*, AbSUR7, the only representative from *A. brassicicola*, showed strong identity (42%) to the *A. nidulans* SurG protein (ANID_04615.1), considered to be the Sur7 orthologue of *S. cerevisiae* and *C. albicans* [36]. The AbNCE102 protein also shared strong identity with Nce102 homologues in *S. cerevisiae* (37% with YPR149W) and *A. nidulans* (42% with ANID_07683). AbSUR7 and AbNCE102 have a typical structure based on four transmembrane domains. AbNCE102 also harbors a Marvel (MAL and related proteins for vesicle trafficking and membrane link) domain, consistent with function related to cholesterol-rich membrane apposition events in a variety of cellular processes. Interestingly we did not find obvious *A. brassicicola* homologues for the following yeast proteins: Fmp45 and Ynl194c (Sur7 family tetraspan), Fhn1 (Nce102 family tetraspan), Rfs1 (flavodoxin-like proteins), Eis1, Seg1, Ygr130c, and Msc3 (all are eisosomal proteins with unknown function).

**Table 1:** Homologous proteins in *A. brassicicola* of the main *S. cerevisiae* MCC/eisosome proteins and their gene expression in response to applied treatments. Brassinin, camalexin, sorbitol and desiccation treatments were compared to non-treated cultures at two time points (0.5 h and 2 h, or 1 h and 4 h). Genes with a *P*-values ≤0.01 and a log2 ratio ≥ 0.5 or ≤ − 0.5 were considered as differentially expressed. Values corresponds to log2 ratio

	Protein	*A. brassicicola* ID	*S. cerevisiae* homolog (% identity)	Function	Reference	Exposure to brassinin		Exposure to camalexin		Exposure to sorbitol		Exposure to dehydration	
Eisosome						**0.5 h**	**2 h**	**0.5 h**	**2 h**	**0.5 h**	**2 h**	**1 h**	**4 h**
	Pil1A	AB08863.1	YGR086C (73%)	BAR domain	[10]	0.88		0.62		2.27	0.88	1.38	1.59
	Pil1B	AB09572.1	YPL004C (50%)	BAR domain	[10]								
	Slm1	AB02000.1	YIL105C (37%)	BAR domain and PH domain	[38]	2.01	0.9	1.6		1.55		2.48	1.74
	Slm2	AB05189.1	YNL047C (24%)	BAR domain and PH domain	[38]							1.18	0.61
	Pkh1	AB04691.1	YDR490C (50%)	Ser/Thr protein kinase	[14, 20]								
	Pkh2	AB07167.1	YGR092W (55%)	Ser/Thr protein kinase	[14, 20]								
	Pst2	AB10448.1	YCR004C (51%)	flavodoxin-like proteins	[36]								
	Mdg1	AB06956.1	YHR146W (38%)	Unknown	[36]	0.85							
	Ycp4	AB02777.1	YCR004C / YDR032C (57%)	flavodoxin-like proteins	[36]						0.79		
	Xrn1	AB10419.1	YGL173C (63%)	Exonuclease	[37]								
MCC	Sur7	AB08885.1	YML052W (34%)	Sur7 family tetraspan	[4, 6]	1.5		0.98		1.72		1.91	1.81
	Pun1	AB03912.1	YLR414C (26%)	Sur7 family tetraspan	[36]					1.39	0.94		
	Nce102	AB04716.1	YPR149W (37%)	Nce102 family tetraspan	[36]	1.59		1.05		1.61		1.61	1.66
	Can1	AB08516.1	YEL063C (43%)	H^+^-driven Arg permease	[6]		−0.96			−0.8	−0.61	− 0.96	
	Fur4	AB04392.1	YIR028W (46%)	H^+^-driven uracil permease	[5]								−1.06
	Tat2	AB08109.1	YKR039W (52%)	H^+^-driven Trp and Tyr permease	[14]

### Expression patterns of a. brassicicola MCC/eisosome components

During leaf and silique infection, *A. brassicicola*, as a necrotrophic agent, is exposed to high levels of antimicrobial defence compounds, such as the indolic phytoalexins brassinin and camalexin. During colonization of maturing seeds, the fungus is exposed to severe water and osmotic constraints [37, 38]. Its ability to cope with such stresses is probably a key factor that determines its aggressiveness and the efficiency of the seed transmission process. To assess if MCC/eisosome may be involved in response to these stresses, we analyzed the MCC/eisosome gene expression in germinated spores of *A. brassicicola* exposed to either indolic phytoalexins brassinin and camalexin (this study) or to water stress (data obtained from [39]). Thus, we produced transcriptome datasets using *A. brassicicola* microarrays bearing one probe for each of the 10,633 ORFs predicted in the *A. brassicicola* automatically annotated genome database. The numbers of genes that were induced or repressed under brassinin or camalexin treatments are shown in the Additional file 2: Figure S1. Global modulation of gene expression in *A. brassicicola* in response to sorbitol and desiccation treatments was previously reported [39].

After exposing germinating conidia to water stresses or indolic phytoalexins, the expression patterns of AbPIL1, AbSLM1, AbSUR7 and AbNCE102 were found to be similar, the corresponding genes being up-regulated at least for one exposure time of each treatment (Table 1). Expression of some genes were induced by only one treatment. For instance, *AbPun1* and *AbYcp4* were induced by the exposure to sorbitol. In contrast, some genes were not differentially regulated (such as the eisosomal protein kinases *AbPkh1* and *AbPkh2*), or were repressed (*AbCan1* and *AbFur4*) by some treatments. Surprisingly, the *AbPil1B* gene, that is considered as a potential paralog of *AbPil1A*, did not show any change in expression.

### Subcellular localization of a. brassicicola MCC/eisosome proteins

**Fig. 2:**
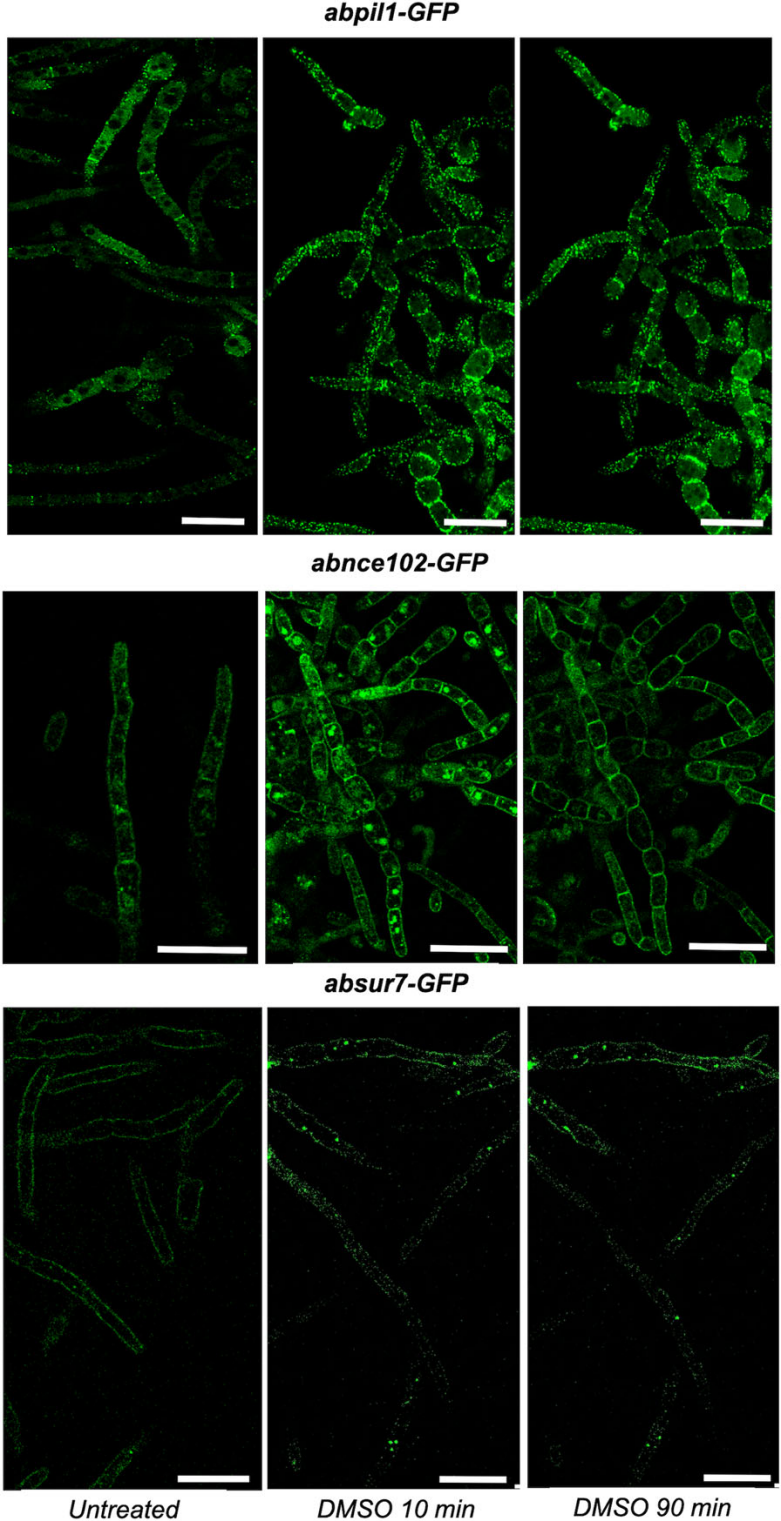
Localizations in *A. brassicicola* germlings of AbPIL1A, AbSUR7 or AbNCE102 fused to GFP fusion proteins. Captures before and after a 90-min period of exposure to 1% DMSO are shown (scale bars = 50 μm)

Strains expressing MCC/eisosome proteins (AbPIL1A, AbSUR7 or AbNCE102, respectively) from their respective endogenous promoter and fused at their carboxy-terminal end to sGFP were engineered. These transformed strains did not show any visible phenotypic changes compared to the wild-type except for expression of green fluorescence (data not shown). As expected, visualization in laser scanning confocal microscopy of hyphal cells showed that AbPIL1A fusion protein tagged by sGFP formed many regular punctuate spots in the PM. These patches were found to be static during a 90-min period of monitoring in the presence or not of DMSO (Fig. 2). In young hyphae, AbNCE102-GFP and AbSUR7-GFP also formed patches in the PM and additionally in intracellular structures. During a 90-min period of growth in the presence of DMSO, this internal staining strongly diminished in the AbNCE102-GFP strain while the peripheric staining remained stable (Fig. 2). The intracellular localizations of these three proteins were consistent with those visualized for the MCC/eisosome proteins in other fungi, such as *A. nidulans*: all showed a PM staining pattern, and NCE102 and SUR7 were additionally detected in other internal structures [39; 42].

### Response of MCC/eisosome mutants to stress conditions

Knockout mutants deficient for AbPIL1A, AbPIL1B, AbSLM1, AbNCE102 (called *∆abpil1a, Δabpil1b, ∆abslm1,* and *∆abnce102*), respectively, were generated. *∆∆abpil1a-abpil1b* double deletion mutants were also constructed. Despite several attempts, we have failed to generate viable *AbSur7* mutants, suggesting that the deletion of this gene in *A. brassicicola* is lethal.

Monitoring growth in standard solid / liquid medium (PDA or PDB) did not reveal any significant effect of MCC/eisosome mutations, compared to the parental strains, on the mycelium growth rate, conidia germination and initial hyphal growth (Additional file 3: Figure S2). We then compared the susceptibility of MCC/eisosome mutants and the wild-type strain to various stress conditions, indicated in Table 2. In general, mutants showed only a few significant differences compared to wild-type. For instance, we did not notice any significant susceptibility to indolic phytoalexins and cell wall stress under these particular growth conditions compared to the wild-type. The most notable difference is the slight hypersensitivity of the *∆∆abpil1a-abpil1b* strain to 2 M sorbitol and to DMSO.

**Table 2:** Susceptibility of *A. brassicicola* wild-type and MCC/eisosome mutant strains to different stress conditions

Applied stress	WT	*∆abpil1a*	*∆abpil1b*	*∆∆abpil1a-abpil1b*	*∆abnce102*	*∆abslm1*
2 M Sorbitol	39.7 ± 1.9	40.8 ± 2.3	44.9 ± 2	51.5 ± 2.2 *	40.3 ± 1.4	45.3 ± 2.9
100 μM Brassinine	40.4 ± 3	39.8 ± 8	38.7 ± 1.6	36.7 ± 1.9	31.1 ± 4.6	33.3 ± 1.5
60 μM Camalexin	66.3 ± 2.8	58.1 ± 1.3	64.9 ± 3.9	61.8 ± 6.4	53.9 ± 1.5	62 ± 1.8
DMSO (1%)	16.5 ± 1.8	19.7 ± 1.36 *	13.9 ± 4.2	27.4 ± 5.2 *	22.1 ± 1.9 *	10.2 ± 5.1
30 mM Menadione	61.6 ± 3.6	61.1 ± 7	62.6 ± 6.8	59.5 ± 2.8	56.3 ± 10	60.7 ± 2.8
400 mg.L^−1^ Calcofluor White	55.3 ± 0.9	52.9 ± 1.5	53.4 ± 1.7	57 ± 4.1	50.3 ± 1.7	55.3 ± 1.8
100 mg.L^−1^ Congo Red	45.7 ± 1.6	42.1 ± 0.9	40.8 ± 5.2	44.9 ± 1.7	40.4 ± 1.1	39.9 ± 4.7

The results are expressed as the percentage of inhibition in treated samples compared to the control without additive. Conidia of each genotype were used to inoculate microplate wells containing standard PDB medium supplemented with the appropriate compound. Nephelometric growth was automatically recorded for 33 h at 24 °C. Each condition was tested in triplicate and the experiments were repeated three times. The areas under the curves were used to calculate the percentages of inhibition for each treatment compared to the control growth curves. Values are means and standard errors of at least nine biological repetitions and represent the percentage growth inhibition under stress conditions compared with standard growth conditions. Conditions denoted with * (*p* ≤ 0.05) were significantly different compared with their respective control

### Investigation of the conidial ultrastructure

**Fig. 3:**
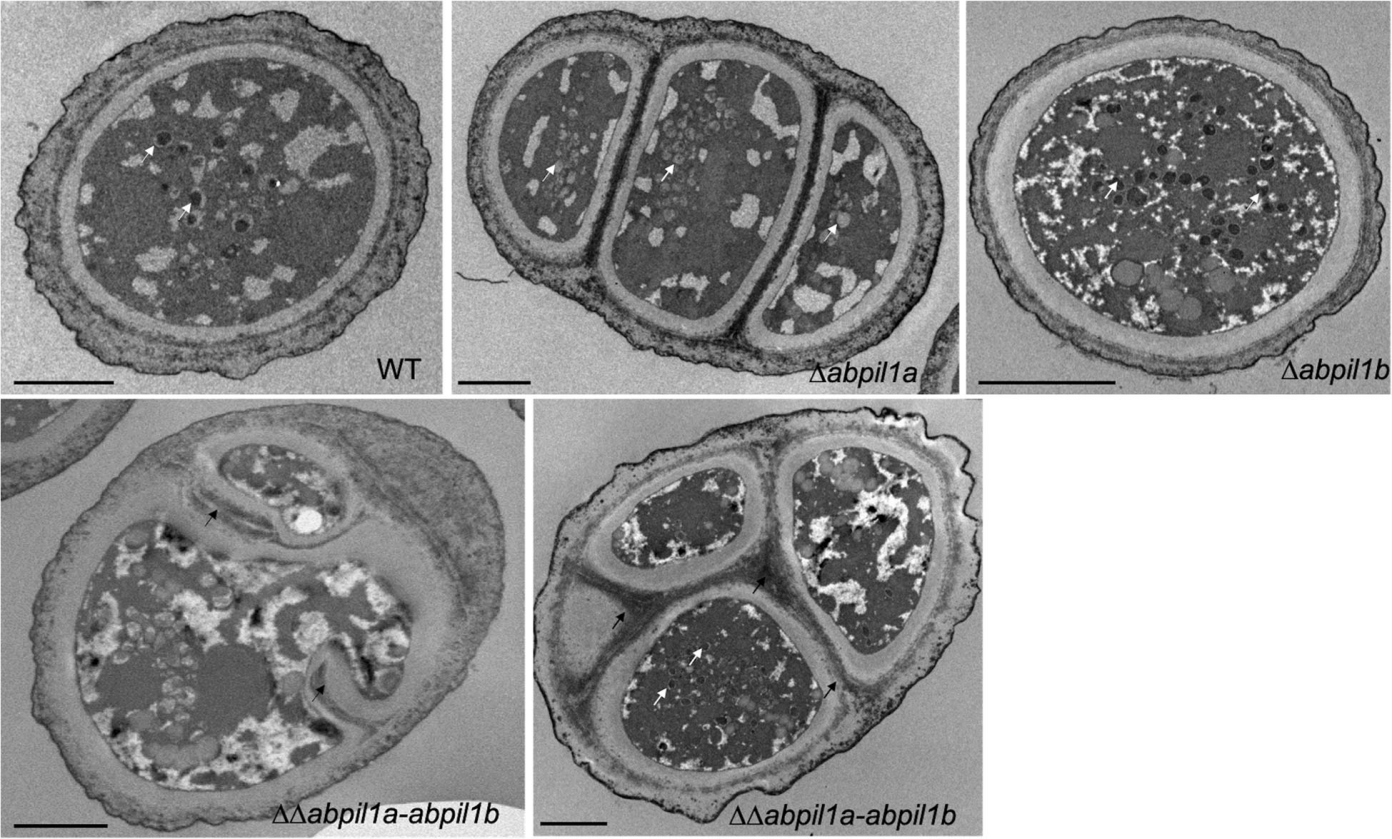
Ultrastructure of *A. brassicicola* conidial cells. Transmission electron micrographs were performed from 7-day-old wild-type (WT) and MCC/eisosome mutant conidia (scale bars = 2 μm). White arrows indicate autophagosomes and black arrows indicate abnormal tubular structures with cell wall material in the middle of the cell

We investigated the morphology of the wild-type and mutant quiescent conidia using transmission electron microscopy (TEM). As shown in the Fig. 3 for the WT and *∆abpil1b* strains, the young spores were small, slightly elongated and without cross-section. Older spores usually had two cross-sections or more (as shown for the *∆abpil1a* strain in the Fig. 3). However, we observed that the cell wall of the double mutant *∆∆abpil1a-abpil1b* formed extensive invaginations. Young spores of the double mutant had indeed one or more septa that were transverse and sometimes longitudinal (Fig. 3). This type of structure was not observed neither in the wild strain nor in the single mutants.

Moreover, the aspect of autophagosomes was examined more specifically since it has already been reported by [29] in *B. bassiana* that deletions of eisosomal genes could impair the formation of intravacuolar autophagosomes. In *A. brassicicola*, the aspect of autophagosomes was also found to be altered in several mutants. The *Abpil1A* mutant did not seem to form autophagosomes or only formed small altered ones in vacuoles. In contrast, in the *∆abpil1b* cells, vacuolar lumens were filled with hyper-developed autophagosomes, which appeared even larger than in wild-type vacuoles (Fig. 3). No specific alteration of autophagosomes was noted in *∆abslm1* and *∆abnce102* cells compared to wild-type cells (data not shown).

### Impact of MCC/eisosome proteins on a. brassicicola virulence

**Fig. 4:**
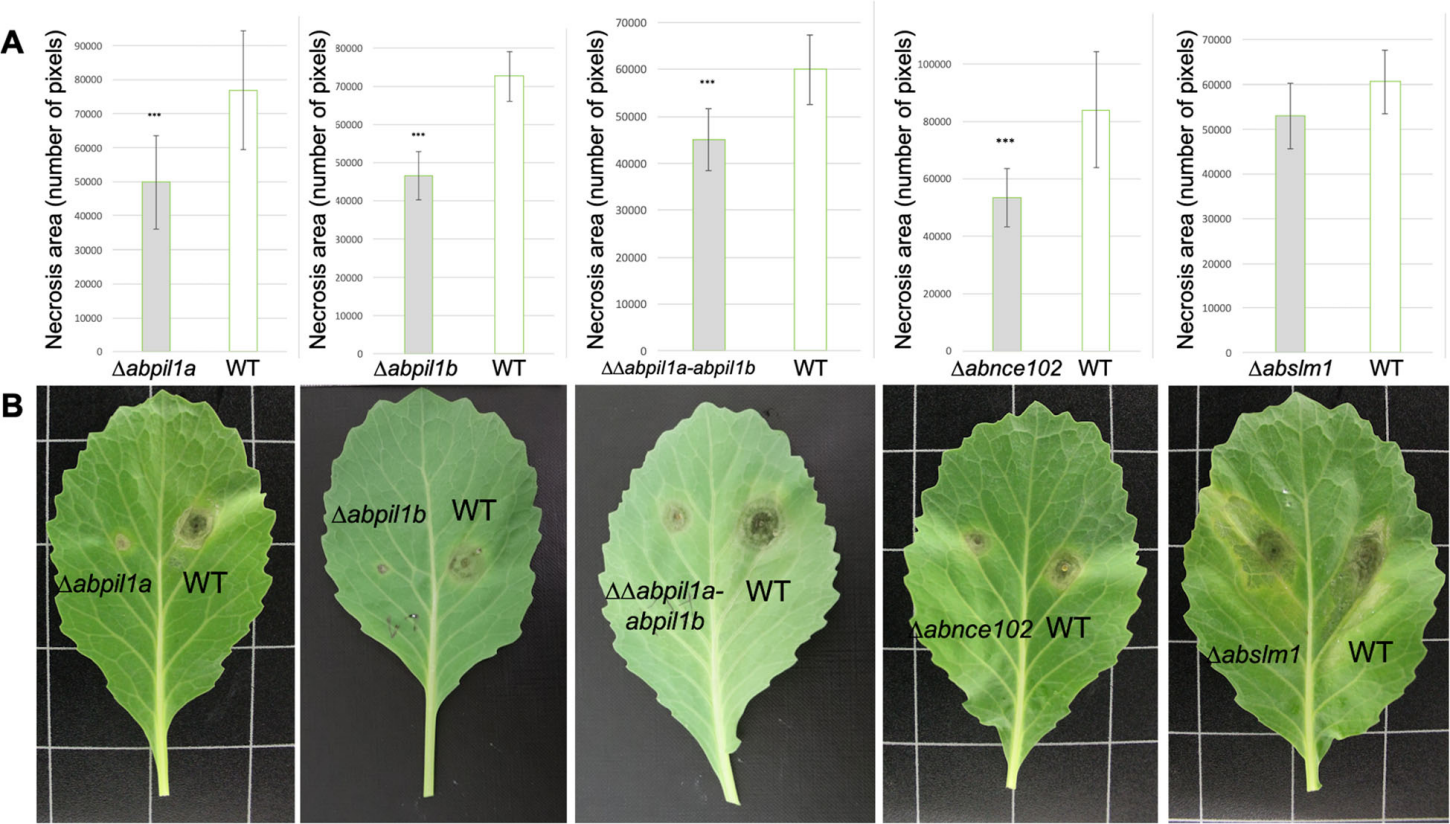
Disease symptoms of *A. brassicicola* on *B. oleracea* leaves. a Mean lesion diameter for all inoculation sites measured at 5 dpi. Values are means ± SEM for at least five replicate experiments. Stars indicate a significant difference between the wild-type and the mutant strains aggressiveness using the paired Wilcoxon test (*P* < 0.05) .b Representative symptoms obtained by inoculation of wild-type (WT) and respective MCC/eisosome mutants at 5 dpi. Leaves were inoculated with conidia suspensions of WT (right part of the central vein) and MCC/eisosome mutants (left part of the central vein) without artificial lesions

Virulence of the wild-type and deficient mutants was compared on *Brassica oleracea* host plants by inoculation experiments on leaves (Fig. 4). All these genotypes were able to produce symptoms, i.e. necrotic areas surrounded by chlorotic halos at six days post-infection (dpi). However, smaller necrotic lesions with very limited spread around the inoculation sites were observed on tissues inoculated with the *∆abpil1a*, *∆abpil1b*, *∆∆abpil1a-abpil1b* and *∆abnce102* mutants, compared to the lesion sizes obtained from the wild-type or *Δabslm1* strains (Fig. 4).

**Fig. 5:**
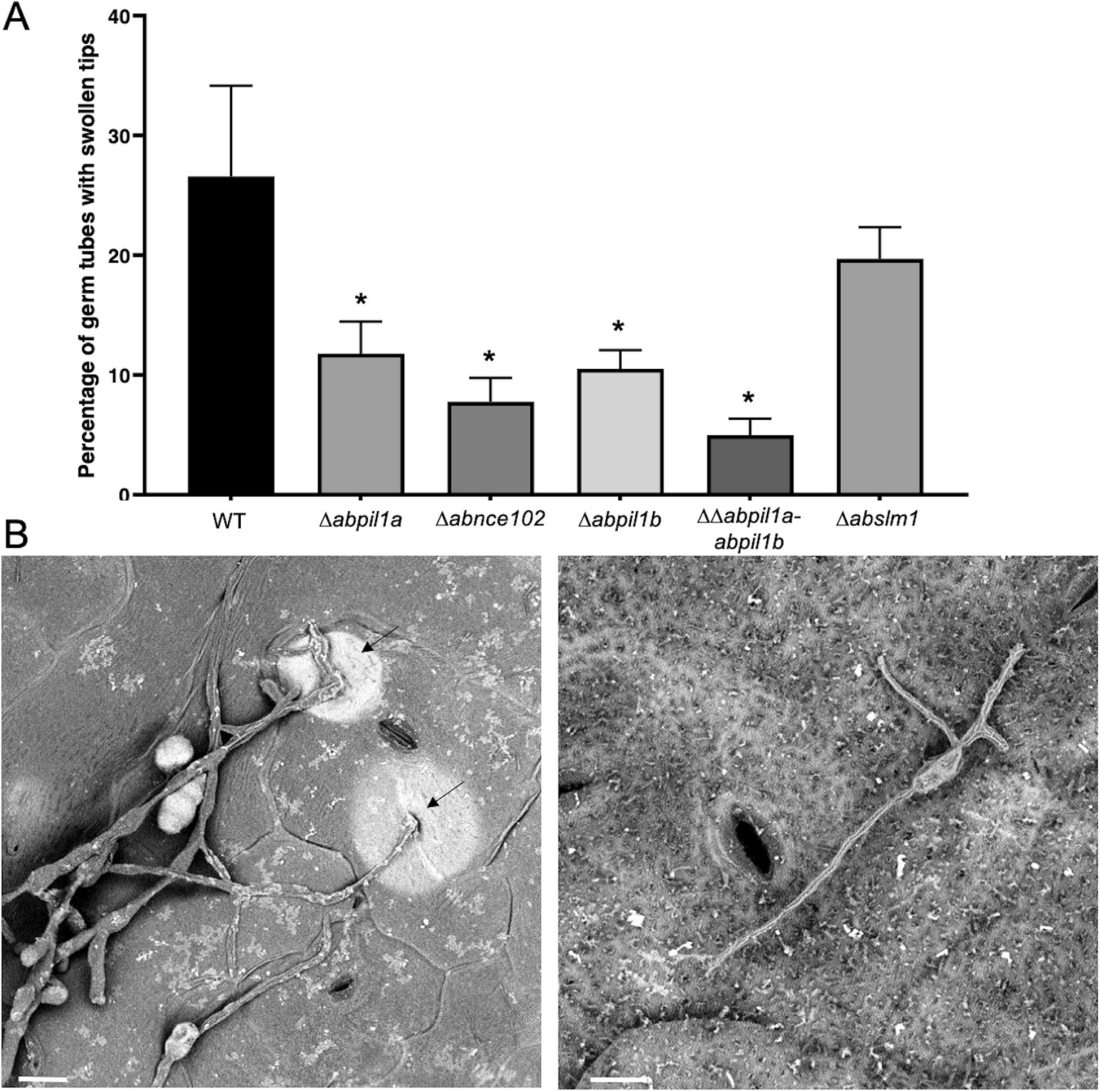
Effects of targeted gene knockout in appressorium-like structure differentiation. a Percentages of germ tubes differentiating swollen tips. Values represent appressorium appearance probabilities with 95% confidence interval. **b** Microscopic observations of the infection structures on *B. oleracea* leaf surfaces. Leaf fragments inoculated with *A. brassicicola* wild-type strain or MCC/eisosome mutants were collected at 24 h dpi and directly imaged with an environmental scanning electron microscope in their natural state without modification or preparation. Appressoria-like structures are indicated by arrows. Scale bars = 10 μm

One day after inoculation, leaves were collected and observed using environmental scanning electron microscopy (SEM). Following spore attachment, we observed that conidia of all deficient mutants germinated with the same efficiency than wild-type conidia on epidermal surface. The emerging germ tubes of fungal necrotrophs often end with appressoria, which are specialised infection structures used to gain entry to plant internal tissues [40]. The germ tube hooks and forms a typical swollen tips from which an infection peg grows and enters the host using turgor pressure and/or enzymatic action. As shown in Fig. 5, more than a quarter of the wild-type germ tubes differentiated swollen tips corresponding to characteristic appressoria-like structures. Moreover, a typical white halo appeared around several appressoria revealing the presence of a functional appressorium that allowed the penetration of the fungus into host tissues. Except for the *Δabslm1* strain, the number of appressorial structures with swollen tips was found to be strongly reduced for other deficient genotypes compared to the wild-type strain, and in particular for *∆∆abpil1a-abpil1b* for which only 5% of germ tubes produced swollen tips.

**Fig. 6:**
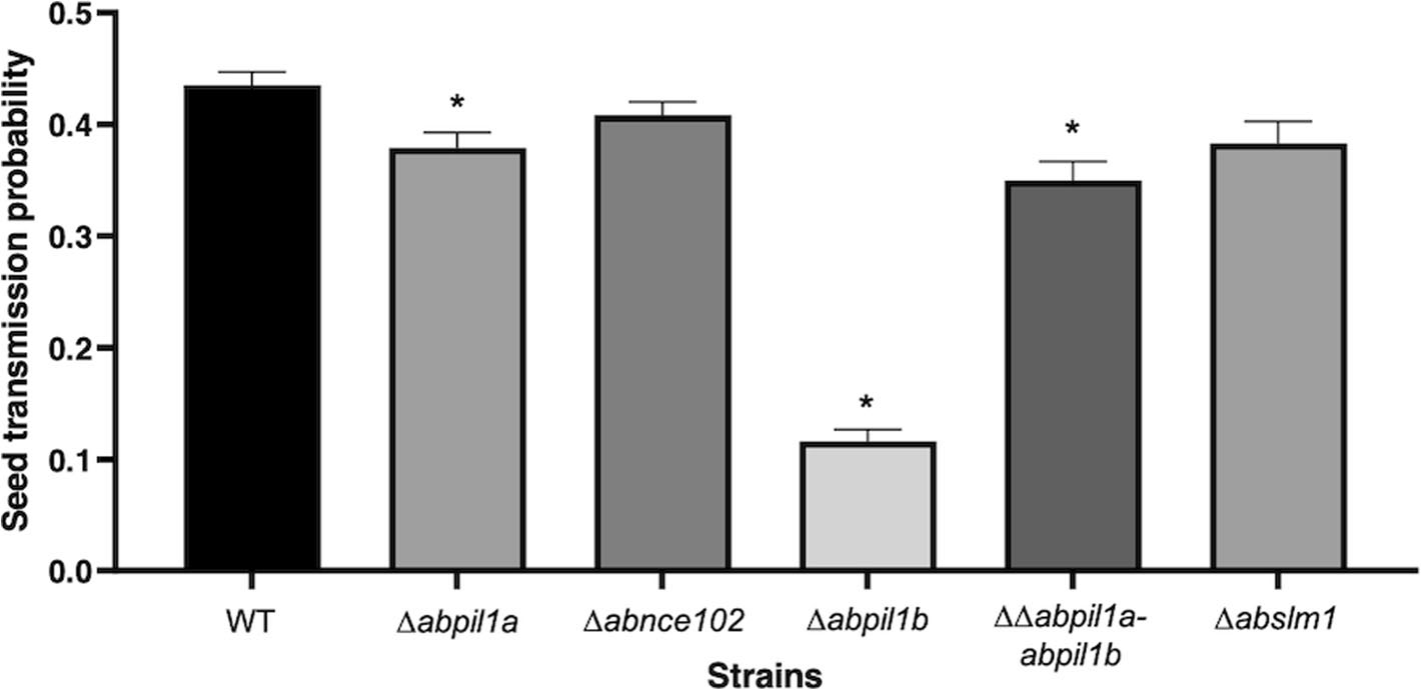
Transmission capacity of *A. brassicicola* wild-type (WT) and MCC/eisosome mutants to *Arabidopsis thaliana* seeds (Ler ecotype). The seed transmission capacity according to the silique stage and global seed transmission capacity (strain model) were measured as described by [37]. The five oldest siliques of at least five plants were inoculated with each fungal genotype and the experiment was repeated . Contaminated siliques were harvested 10 dpi. After dissection, seeds were incubated separately on PDA medium for 2 days. A seed was considered contaminated when incubation resulted in typical *A. brassicicola* colony development. For each inoculated fungal genotype, the seed infection probability was evaluated from at least 1000 seeds. Values represent infection probabilities with 95% confidence interval

Using the model pathosystem previously described by [37] for investigating *A. brassicicola* seed transmission in *Arabidopsis thaliana Ler ecotype*, the ability of the MCC/eisosome mutants to transmit to seeds was compared with that of the wild-type. Silique inoculation with the wild-type and mutant strains resulted in the onset of necrotic lesions typical of black spot on siliques within a few days after inoculation. However, a significant decrease in global seed transmission was observed for the *∆abpil1a*, *∆abpil1b*, *∆∆abpil1a-abpil1b* deletion mutants compared to the wild-type (Fig. 6). No significant transmission alteration was observed for *∆abnce102* and *Δabslm1* strains.

## Discussion

The *S. cerevisiae* MCC/eisosome protein sequences were used as probes to identify homologues in the *A. brassicicola* genome. We identified ten *A. brassicicola* eisosomal proteins, including AbPIL1A and AbPIL1B, and six others MCC proteins, including AbSUR7 and AbNCE102.

The intracellular localizations of AbPIL1A, AbSUR7 or AbNCE102 were investigated by expressing respective GFP fusion proteins from their respective endogenous promoter. In *A. brassicicola*, the intracellular localizations of these three proteins were consistent with those visualized for the MCC/eisosome proteins in yeasts and other filamentous fungi. First, deconvoluted Z stacks of AbPIL1A and time-lapse live-cell imaging in quiescent conidia and germlings showed stable punctuate spots with a plasma membrane staining pattern, as previously observed in *S. cerevisiae*, *C. albicans*, *Ashbya gossypii*, *A. nidulans* and *B. bassiana* [10, 29, 36, 41, 42]. As expected, AbSUR7-GFP localized to static patches in the PM but a punctuate intracellular fluorescence signal was also observed in hyphae. This is consistent with the localization pattern of SurG-GFP in *A. nidulans*, that was found as patches at the cell periphery of the spores and was additionally localized perinuclearly [36]. In germlings, AbNCE102 showed a PM staining pattern, consistent with a MCC/eisosome localization, and was additionally detected in other intracellular structures, that could be endoplasmic reticulum (ER) and vacuoles, as previously reported in yeast and *A. nidulans*. In *A. nidulans*, a co-localization of AnNce102 with the chaperone ShrA from the ER and with the vacuolar tracer CMAC was reported [43]. Here, using time-lapse live-cell imaging during a 90-min period of growth in the presence of DMSO, we showed that AbNCE102 formed stable spots with low mobility at the PM. On the contrary, the staining of internal organelles strongly diminished [43]. also showed that the spatial AnNce102 distribution could fluctuate during germination of *A. nidulans*. In parallel, we also generated a *abpil1b-GFP* strain but the resulting fluorescence signal was almost undetectable into the fungal cells and was too weak to investigate a possible co-localization of AbPIL1A with AbPIL1B, as it was reported in other fungi. This low level of fluorescence might be due to a low level of the AbPIL1B protein compared to AbPIL1A, as already suggested from our transcriptomic analysis, or to an artifactual event during the construction of the transgenic strain.

Although *abpil1a*, *abnce102* and *absur7* were found to be over-expressed after exposing germinating conidia to water stresses or indolic phytoalexins, the respective deficient strains did not exhibit any significant susceptibility to various stress conditions, with the exception of a slight hypersensitivity of mutant strains to 2 M sorbitol *(∆∆abpil1a-abpil1b*) and to 1% DMSO (*∆∆abpil1a-abpil1b, ∆abpil1a, ∆abnce102*). Compared to wild-type, the most noticeable differences concerned the conidial ultrastucture. As reported in *ΔΔpil1lsp1* and *Δsur7* cells of *C. albicans* [44] and in *Δpil1* cells of *S. cerevisiae* [10], *∆∆abpil1-ablsp1* cell walls formed extensive invaginations [44]. suggested that these abnormal cell wall phenotype were probably linked to abnormal regulation of PI4,5P_2_, since similar broad invaginations of cell wall material were observed in a *C. albicans ∆inp51* mutant or in an *S. cerevisiae ∆∆inp51-inp52* mutant, which lacks PI5-specific PI4,5P_2_ phosphatases [44–46]. Consistently, they showed that sites of altered cell wall growth were associated with elevated levels of PI4,5P_2_ in *ΔΔpil1-lsp1* and *Δsur7* [44]. Interestingly, compared to wild-type, *A. brassicicola* MCC/eisosome mutants did not exhibit thicker cell walls and did not show hypersensitivity to cell wall stress, as it was reported in other fungi [29, 44]. We also examined the aspect of autophagosomes in *A. brassicicola* MCC/eisosome mutants and observed that *∆abpil1a* and *∆abpil1b* mutants failed to form normal autophagosomes. In fungi, autophagy mediates the incorporation of the cytoplasm and organelles into vacuoles or lysosomes for degradation. This process, which is negatively regulated by the phosphatidylinositol kinase-related kinase Tor in yeast [47], plays an important role (i) in maintaining cellular homeostasis by allowing the elimination and continuous replacement of non-functional proteins and organelles and (ii) in promoting adaptation and survival of cells under stress conditions [48, 49] [29]. previously observed the enlargement and abolishment of autophagosomes in *B. bassiana Δpil1A* and *Δpil1B*, respectively. They also reported that deletions of *pil1A* and *pil1B* caused opposite changes in expression of many autophagy-related genes and that nearly basal expression patterns were restored by application of exogenous rapamycin [29]. All these results suggested a strong link between the target of rapamycin (TOR) signaling pathway and MCC/eisosome for the completion of the autophagic process in filamentous fungi.

For the first time, the role of MCC/eisosomes in the pathogenic process of a plant pathogenic fungus was investigated. *A. brassicicola* is a necrotrophic fungus causing black spot disease in a wide range of Brassicaceae plants. Contrary to biotrophic pathogens that derive nutrients from living host tissues, *A. brassicicola* kills and absorbs nutrients from dead or dying cells of aerial parts of its hosts. Once the fungus has entered plant tissue, it grows subcuticularly and kills epidermal cells by secreting toxic metabolites, although no host-specific toxins have been identified so far in this fungus and that some of its pathogenesis mechanisms are still unclear [50]. In this study, we showed that, with the exception of *Δabslm1,* the MCC/eisosomal mutants had reduced virulence on *B. oleracea* leaves. Since these mutants do not exhibit particular vulnerability to phytoalexin or oxidative stress, we hypothesize that their impaired virulence is mainly due to a very low appressorium formation rate. Appressoria are usually differentiated in response to chemical (such as epicuticular waxes and cutin monomers) or physical (hydrophobicity, thigmotropism) cues [40, 51]. Unlike the model fungus *Magnaporthe oryzae* which accumulates glycerol as a solute within the appressorium to generate enormous cellular turgor [52], *A. brassicicola*, as most necrotrophs do, forms inconspicuous appressoria. It probably penetrates the plant cuticle by secreting large amounts of plant cell wall–degrading enzymes from these specialised infection structures. However, it is not clear whether the build-up of large turgor pressure also occurs in *A. brassicicola* appressoria. The presence of symptoms, even in mutants that produce fewer appressoria, was probably due to the ability of the fungus to alternatively penetrate through natural openings, such as stomata or wounds, as previously described by [37]. For all the MCC/eisosome mutants and wild-type strains, occasional penetrations of hyphae through stomata were indeed observed,

Based on the extensive works (in particular in *M. oryzae*) aimed at identifying the cellular and molecular mechanisms involved in appressorium generation [40, 53], we can put forward several hypotheses to explain the alteration of appressoria production in MCC/eisosome mutants. First, we can guess that the localization of actins and septins is probably altered in *A. brassicicola* MCC/eisosome mutants. A first clue consistent with this hypothesis is that some *S. cerevisiae* septin mutants formed abnormal cell wall invaginations [54] as seen in *A. brassicicola ∆∆abpil1a-abpil1b*. Septins are cytoskeletal GTP-binding proteins that were first described as involved in bud emergence in yeast by assembling into filaments and forming a ring on the inner surface of the plasma membrane [55]. Septins are also able to interact with BAR proteins (such as Pil1 and Lsp1) involved in membrane curvature generation [56]. In addition to cytokinesis, they are essential for other critical cellular processes, such as spatial compartmentalization and polar growth, and act as membrane diffusion barriers. As a consequence, septins were shown to play a major role in fungal pathogenesis, in particular by driving the invasive growth [57]. In *M. oryzae*, a hetero-oligomeric septin ring forms at the base of the *appressorium* and scaffolds F-actin via phosphatidylinositide interactions. This ring acts as a diffusion barrier to localise proteins involved in polarity and membrane curvature generation, such as BAR proteins, leading to the extension of a rigid penetration hypha able to rupture plant cuticles [56]. Even if the functioning of *A. brassicicola* appressoria must partly differ from that of *M. oryzae* appressoria, it appears possible that mutations of MCC/eisosome components altered the *A. brassicicola* capacity to focus the force at a single point for tissue invasion. In *C. albicans*, ∆*sur7* and *∆nce102* mutants also exhibited a defect to form invasive hyphae, which is partly due to a partial defect in actin organization and mislocalization of septins and actin [58]*,* and showed a greatly reduced virulence in a mouse model of systemic infection [30, 31].

MCC/eisosome components were also linked to some signaling kinases that were shown to be involved in appressorium formation and pathogenicity in several plant pathogens. For instance, connections between eisosomes and the mitogen-activated protein kinase (MAPK) pathways mediated by Slt2/Mps1 and Fus3/Pmk1 were reported [20, 23, 27]. In *A. brassicicola*, [59] showed that the AbSLT2-mediated cell wall integrity pathway was essential for full expression of fungal virulence. The *∆abslt2* mutant produced less appressoria than the reference strain and consequently was impaired in its tissue penetration capacity [59]. The Pmk1 MAPK pathway was also shown to be essential for appressorium formation and invasive growth in *M. oryzae* [60] and in other plant pathogenic fungi that do not use physical force to break the plant cuticle, such as *Claviceps purpurea*, *Mycosphaerella graminicola*, and *Stagonospora nodorum* [61]. In *A. brassicicola*, the *Pmk1* homolog (*Amk1*) was also essential for full virulence and the *amk1* mutant produced a majority of swollen and immature appresorium-like structures [62]. Moreover, an increasing body of evidence suggests the existence of crosstalk in *M. oryzae* between the Pmk1 MAPK pathway and autophagy, which is also an essential pathway for appressorium formation [63]. According to these results, we can speculate that the defect of appressorium formation in MCC/eisosome mutant domains was caused, at least in part, by actin/septin mislocalisation and/or alteration of the autophagic process and/or improper regulation of MAPK cascades in these specialised infection structures. Next steps of this work will be to validate the potential involvement of these molecular factors in the appressorium formation.

## Conclusions

In this study we identified several potential MCC/eisosomal protein encoding genes in the plant pathogenic fungus *A. brassicicola.* As expected, the PM is the primary localization of these proteins in the *A. brassicicola* cells. As previously shown in other fungi, the deletion of *pil1* and/or *lsp1* homologs altered the conidial cell wall phenotype as well as the aspect of autophagosomes. While the growth of deficient strains was not or only slightly affected by exposure to various stresses, we found that most of the MCC/eisosomal mutants had reduced virulence on *B. oleracea* leaves and were impaired in their capacity to efficiently colonize *A. thaliana* seeds. Since these mutants exhibited a very low appressorium formation rate, we hypothesized that their defect in aggressiveness was caused, at least in part, by a reduced capacity to gain entry to plant internal tissues. This study suggests that interfering with integrity and functions of potential MCC/eisosome domains would be an effective plant disease control strategy.

## Methods

### Strains and culture conditions

The *A. brassicicola* wild-type strain *Abra43* used in this study was previously described [64]. For routine culture, *A. brassicicola* was grown and maintained on potato dextrose agar (PDA).

Concerning the phenotyping of wild-type and mutant strains, the sorbitol assays were carried out on PDA plates at 24 °C supplemented or not with 2 M sorbitol and incubated for 10 days. In all cases, colony diameters were measured and used for calculation of radial growth. All the other assays were carried out in liquid media. Conidial suspensions (10^5^ conidia/mL, final concentration) were inoculated onto microplate wells containing the considered substance at the desired concentration in potato dextrose broth (PDB, Difco) in a total volume of 300 Μl*. medium* was thus supplemented with brassinin (100 μM), camalexin (60 μM), Congo Red (100 mg/mL), Calcofluor White (400 mg/mL), dimethyl sulfoxide (DMSO, 1%), and menadione (30 mM), respectively. Brassinin was synthesized according to [65, 66] while camalexin was synthesized according to [67]. The solvent use for camalexin and brassinin was DMSO and the concentrations in controls and assays did not exceed 0.5% (v/v). Microplates were placed in a laser-based microplate nephelometer (NEPHELOstar, BMG Labtech) and growth was monitored automatically over a 30 h period. Each condition was tested at least in triplicate and the experiments were repeated three times. Nephelometry, which is based on scattered light measurements, was proved to be an accurate indicator of the fungal biomass and can be used as reliable tool for fungal growth monitoring [68]. Data were exported from Nephelostar Galaxy software and further processed in R Studio. Under curve area were calculated and analyzed from the growth curves.

For transcriptomic analyses, brassinin (100 μM) or camalexin (50 μM) were added to 1-day-old germinating conidia (10^5^ conidia.mL^− 1^, final concentration) grown in potato dextrose broth (PDB). Incubation was then carried out for 0.5 and 2 h at 24 °C. Controls were performed by adding DMSO (1% v/v final concentration) instead of the tested compound. Sorbitol and desiccation stresses were achieved either by exposing the fungal cultures to 1.2 M sorbitol for 0.5 h and 2 h (osmotic stress) or by applying to the germlings a drastic decrease in relative humidity by exposure to silica gel beads in sealed boxes for 1 h and 4 h (dehydration stress), as described by [39]. Conidia were first incubated for 24 h on cellophane disks overlaid on solid PDA medium. Germlings and the underlying cellophanes were then taken off from the agar plate and transferred either to a second PDA medium supplemented or not with 1.2 M sorbitol, or in sealed boxes with or without silica gel beads.

### Generation of targeted gene replacement constructs and fungal transformation

The gene replacement cassettes were generated using the double-joint PCR procedure [69] as described by [39] and with primers presented in the Additional file 1: Table S1. They contained the 5′ and 3′ flanking regions of each targeted gene fused with the *Hph* gene cassette (1436 bp) from pCB1636 [70] or the *Nat* gene cassette (2150 bp) from pNR [71] conferring resistance to hygromycin B and nourseotricin, respectively. Knockout mutants deficient for AbPIL1A, AbSLM1, AbNCE102 (called *∆abpil1a, ∆abslm1,* and *∆abnce102*), respectively, were constructed by replacing the respective ORFs with a hygromycin B resistance cassette while *Δabpil1b* strains were generated using a nourseothricin resistance cassette. The *∆abpil1a* genotype was used to obtain *∆∆abpil1a-abpil1b* hygromycin B and nourseotricin resistant strains. The double-joint final PCR products were transformed into *A. brassicicola* protoplasts as described by [72]. For each targeted gene, three replacement mutants were selected using a PCR screen (Additional file 4: Figure S3) and purified by two rounds of single-spore isolation. In all further experiments, the phenotypic characters for the three transformants of the same genotype were not found to be significantly different and the results presented here correspond to means of values obtained for individuals carrying the same mutation.

### Generation and examination of fusion protein constructs

The *Abpil1a*, *Abpil1b*, *AbSur7* and *AbNce102* C-terminal GFP fusion constructs were generated by fusion PCR as described by [73]. Using *A. brassicicola* genomic DNA as a template, the respective ORFs and 3′ flanking regions were amplified with relevant primer combinations (Additional file 1: Table S1). In parallel, a fragment containing the *GFP* cassettes and *HygB* cassettes were amplified from the plasmid pCT74 [74]. The resulting PCR fragments were mixed and subjected to second fusion PCR. A linker containing three glycine residues was introduced at the 3’end of the respective ORFs to replace the stop codons. The final PCR products were transformed in the *A. brassicicola* wild-type as described above. The transformants with expected genetic integration events were identified by PCR. Observations were performed under a Nikon (Nikon Instruments, Melville, NY) A1S1 confocal laser microscope equipped with argon-ion (488 nm) and diode (561 nm) lasers.

### Electron microscopy

The fungal cell ultrastructure was investigated by TEM (transmission electron microscopy) using conidial suspensions obtained from 7-day-old cultures on PDA. The sample fixation was done by 2% glutaraldehyde and 2% perfluoroalkoxy buffered at pH 7.4 with 0.1 M sodium cacodylate at night under vacuum at room temperature. Fungal cells were postfixed for 1 h in OsO_4_ 1%, dehydrated in ethanol and embedded in Epon. Thin sections were contrasted with uranyle acetate examined under a JEM-1400 transmission electron microscope (Jeol, Paris, France).

SEM (scanning electron microscopy) pictures were acquired with the Phenom Pro desktop SEM (Phenom-World). The samples were directly imaged in their natural state without modification or preparation. Appressorium formation data were obtained using logistic (logit) generalized linear models as previously described [37].

### Gene expression monitoring

Total RNA extraction and microarray analysis were performed as described by [39]. The Abra_v1.0 chip (Nimblegen, Madison, WI) contained 10,633 60-mer oligoprobes that were designed from the *A. brassicicola* genome database (https://genome.jgi.doe.gov/Altbr1/Altbr1.home.html). Three biological replicates were analyzed per comparison using the dye-switch method. Only probes with a *P*-values ≤0.05 and a log2 ratio ≥ 0.5 or ≤ − 0.5 were considered as differentially expressed. Gene expression datasets were deposited in the Gene Expression Omnibus (GEO) with the following accession numbers, GE140386 and GSE133507.

### Infection assays

Plant infection assays were performed on *Brassica oleracea* (var. Bartolo), as previously described [59]. Symptoms were observed at 5 days post-inoculation (dpi). The experiment was repeated 5 times and for each experiment each genotype was inoculated onto 9 leaves.

Silique infection assays and seed contamination assessments were performed as previously reported [37]. *A. thaliana* L*er* seeds were provided by the Nottingham Arabidopsis Stock Center.

## Supplementary information

**Additional file 1: Table S1:** List of primers used in this study.

**Additional file 2: Figure S1:** Venn diagrams showing global modulation of gene expression in *A. brassicicola* in response to brassinin and camalexin treatments. Phytoalexin treatments were compared to non-treated cultures at two time points 0.5 h and 2 h. Genes with a *P*-values ≤0.05 and a log2 ratio ≥ 0.7 or ≤ − 0.7 were considered as differentially expressed.

**Additional file 3: Figure S2:** Representative growth of wild-type (WT) and respective MCC/eisosome mutants. **a** Radial growth in standard solid medium (PDA) after incubation at 22 °C for 5 days. **b** Growth curves monitored in a laser-based microplate nephelometer over a 30 h period in standard liquid medium (PDB).

**Additional file 4: Figure S3:** Generation of *∆abpil1a, ∆abpil1b, ∆abslm1,* and *∆abnce102* by homologous recombination. **a** Schematic representation of a targeted locus in WT and in mutant strains after integration of the replacement construct containing the hygromycin B (*Hph* gene) or the nourseothricin (*Nat* gene) resistance cassette and flanking sequences. Primers used for PCR screening of mutants are indicated. **b** Gel electrophoresis of PCR products obtained from template DNA of the WT, *∆abpil1a, ∆abpil1b, ∆abslm1,* and *∆abnce102* strains with the indicated primer pairs. Molecular sizes (kb) were estimated based on a 1 kb ladder (lane L2, New England Biolabs) or SmartLadder (Lane L1, Eurogentec). ITS1/4 primers were used as a positive control for PCR.

## Data Availability

Gene expression datasets were deposited in the Gene Expression Omnibus (GEO) (https://www.ncbi.nlm.nih.gov/geo/query/acc.cgi?acc=GSE133507 and https://www.ncbi.nlm.nih.gov/geo/query/acc.cgi?acc=GSE140386). The biological material used and analyzed during the current study is available from the corresponding author on reasonable request.

## Abbreviations

AH domain: Arfaptin homology domain
BAR domain: Bin/Amphiphysin/Rvs domain
DMSO: dimethyl sulfoxide
dpi: days post-infection
ER: Endoplasmic reticulum
GFP: Green fluorescent protein
JGI: Joint genome institute
MAPK: Mitogen-activated protein kinase
MCC: Membrane compartment of Can1
MCP: Membrane compartment Pma1
MCT: Membrane compartment of TORC2
MCW: Membrane compartment occupied by Wsc1
ORF: Open reading frame
PCR: Polymerase chain reaction
PDA: Potato dextrose agar
PDB: Potato dextrose broth
PI4,5P_2_: Phosphatidylinositol 4,5- bisphosphate
PM: Plasma membrane
SEM: Scanning electron microscopy
TEM: Transmission electron microscopy
TOR: The target of rapamycin
WT: Wild-type
