# Supplementary figures and images for: Role of membrane compartment occupied by Can1 (MCC) and eisosome subdomains in plant pathogenicity of the necrotrophic fungus Alternaria brassicicola
Source: BMC Microbiol. 2019 Dec 16;19:295. doi: 10.1186/s12866-019-1667-4 (PMC6916069; doi:10.1186/s12866-019-1667-4)

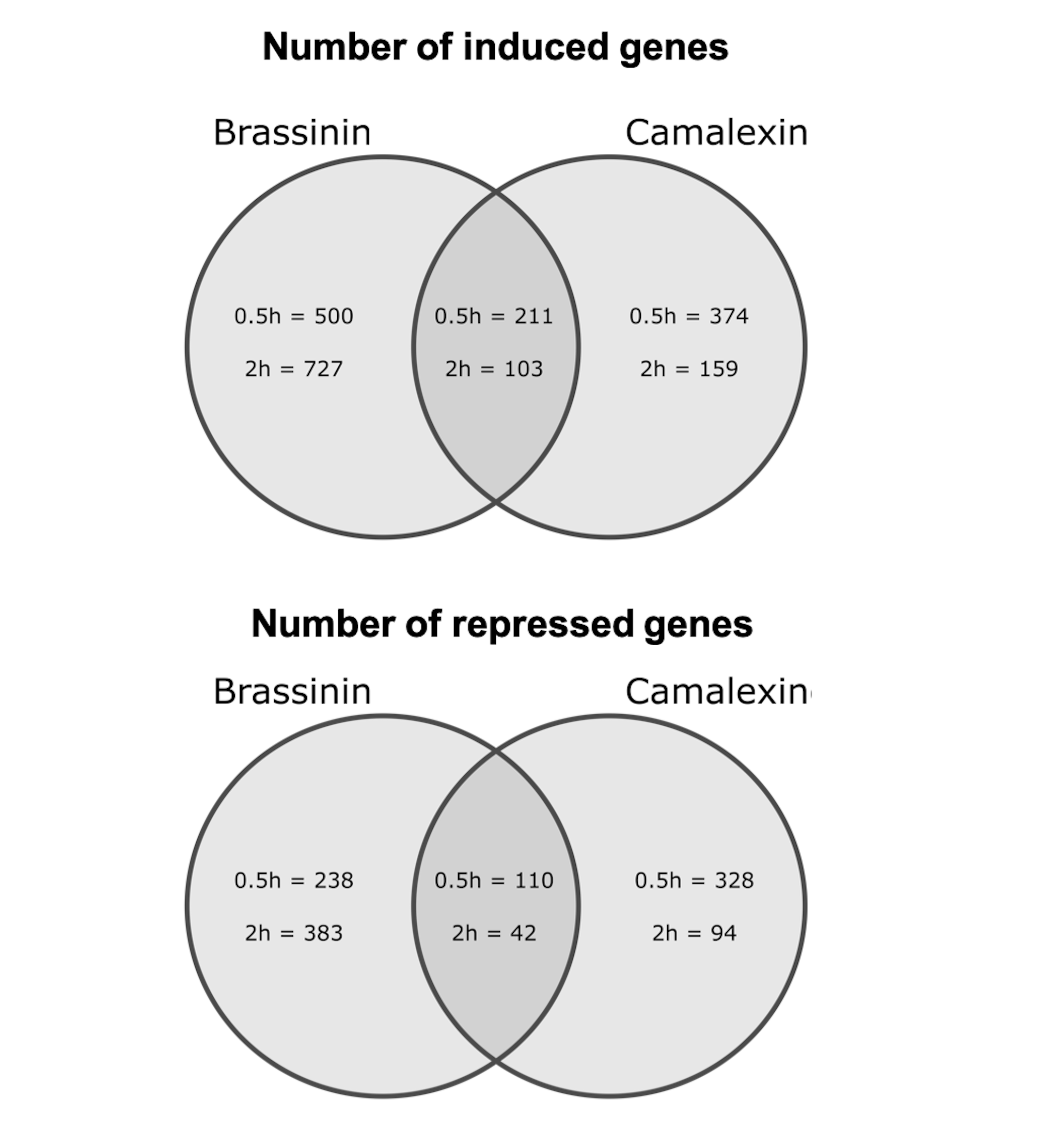

Supplement: Supplementary file 2 — Additional file 2: Figure S1: Venn diagrams showing global modulation of gene expression in A. brassicicola in response to brassinin and camalexin treatments. Phytoalexin treatments were compared to non-treated cultures at two time points 0.5 h and 2 h. Genes with a P-values ≤0.05 and a log2 ratio ≥ 0.7 or ≤ − 0.7 were considered as differentially expressed. [file 12866_2019_1667_MOESM2_ESM.png]

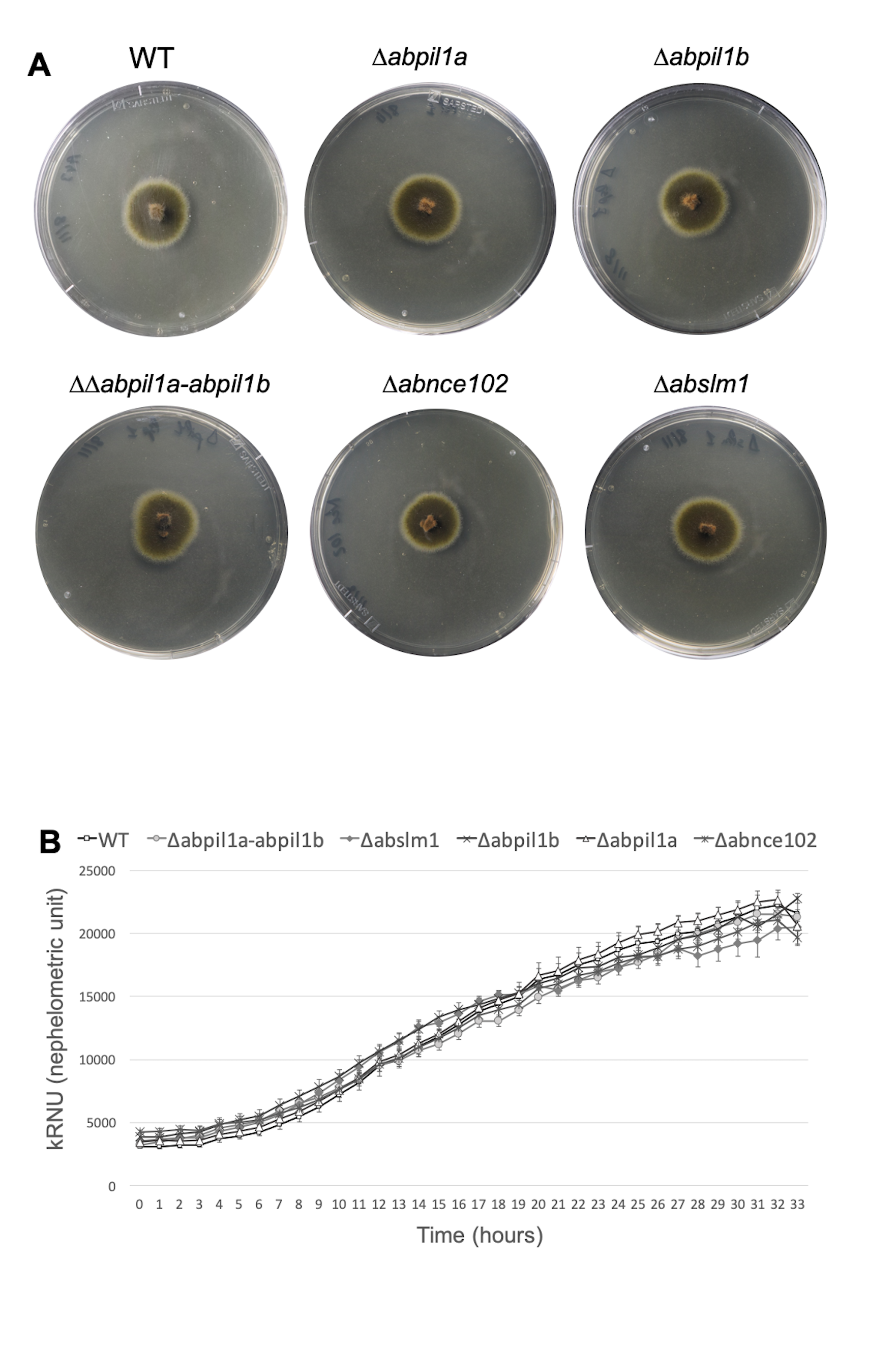

Supplement: Supplementary file 3 — Additional file 3: Figure S2: Representative growth of wild-type (WT) and respective MCC/eisosome mutants. a Radial growth in standard solid medium (PDA) after incubation at 22 °C for 5 days. b Growth curves monitored in a laser-based microplate nephelometer over a 30 h period in standard liquid medium (PDB). [file 12866_2019_1667_MOESM3_ESM.png]

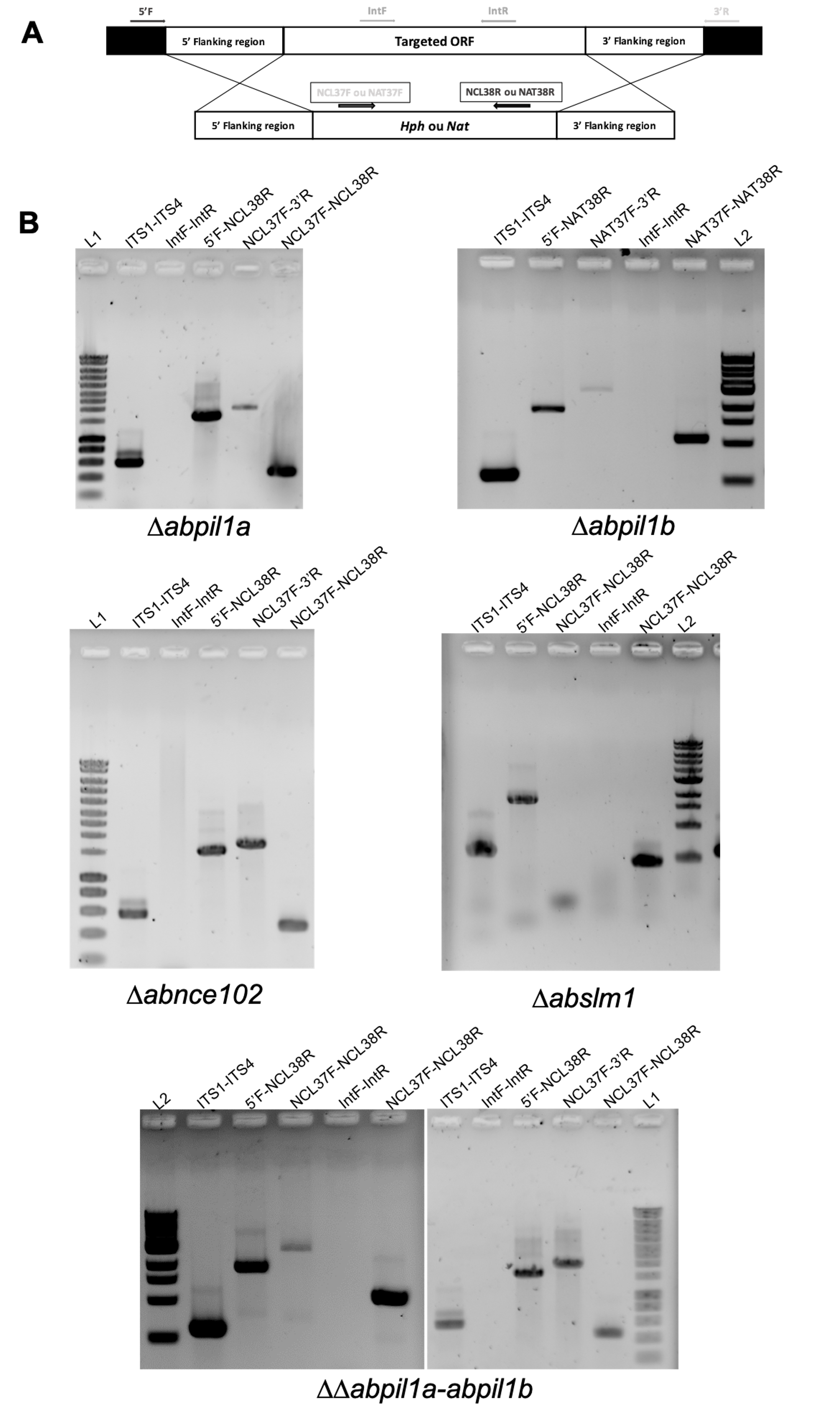

Supplement: Supplementary file 4 — Additional file 4: Figure S3: Generation of ∆abpil1a, ∆abpil1b, ∆abslm1, and ∆abnce102 by homologous recombination. a Schematic representation of a targeted locus in WT and in mutant strains after integration of the replacement construct containing the hygromycin B (Hph gene) or the nourseothricin (Nat gene) resistance cassette and flanking sequences. Primers used for PCR screening of mutants are indicated. b Gel electrophoresis of PCR products obtained from template DNA of the WT, ∆abpil1a, ∆abpil1b, ∆abslm1, and ∆abnce102 strains with the indicated primer pairs. Molecular sizes (kb) were estimated based on a 1 kb ladder (lane L2, New England Biolabs) or SmartLadder (Lane L1, Eurogentec). ITS1/4 primers were used as a positive control for PCR. [file 12866_2019_1667_MOESM4_ESM.png]
